# Dietary Zinc Acts as a Sleep Modulator

**DOI:** 10.3390/ijms18112334

**Published:** 2017-11-05

**Authors:** Yoan Cherasse, Yoshihiro Urade

**Affiliations:** International Institute for Integrative Sleep Medicine (WPI-IIIS), University of Tsukuba, 305-8575 Tsukuba, Japan; urade.yoshihiro.ft@u.tsukuba.ac.jp

**Keywords:** sleep, zinc, nutrition, brain, randomized controlled trial

## Abstract

While zinc is known to be important for many biological processes in animals at a molecular and physiological level, new evidence indicates that it may also be involved in the regulation of sleep. Recent research has concluded that zinc serum concentration varies with the amount of sleep, while orally administered zinc increases the amount and the quality of sleep in mice and humans. In this review, we provide an exhaustive study of the literature connecting zinc and sleep, and try to evaluate which molecular mechanism is likely to be involved in this phenomenon. A better understanding should provide critical information not only about the way zinc is related to sleep but also about how sleep itself works and what its real function is.

## 1. Introduction

Zinc is the second most abundant trace metal in the human body, and is essential for many biological processes. Nevertheless, many new functions remain to be discovered for this unique divalent cation. A very recent body of evidence suggests that zinc is involved in the regulation of sleep, one of the most essential physiological functions in the entire animal kingdom. The aim of this review is to provide an overview of the current data suggesting that zinc influences sleep in mice and humans. We first provide a brief background on what sleep is, and what we know of its regulation. We then explore the identified roles of zinc in the brain, as well as how food regulates sleep. Furthermore, we perform a comprehensive review of the literature connecting zinc and sleep. Finally, we discuss possible molecular mechanisms by which zinc can act in the brain and regulate sleep.

## 2. Sleep

Sleep is defined as a natural periodic state of rest, in which the eyes remain closed and consciousness is completely or partially abolished, so that there is a decrease in bodily movement and responsiveness to external stimuli. However, an important aspect of sleep, unlike coma or anesthesia, is that it must be easily and immediately reversible. While everybody experiences sleep, 2500 years of research since Alcmaeon, Hippocrates and Aristotle (450–350 B.C.) could not yet clearly elucidate the most simplest question as “why do we need to sleep?”. To finally answer this question, today’s sleep research efforts are mostly focused on understanding how sleep is regulated.

Sleep is a process common to the whole animal kingdom, from *Caenorhabditis elegans* to *Drosophila melanogaster*, zebrafish and of course mammals [1]. Sleeplessness has a huge impact on the human physiology and is commonly associated with metabolic disorders (obesity and diabetes), cardiovascular diseases (hypertension) and mental disorders (anxiety and depression). Furthermore, insomnia has also recently been associated with neurodegenerative diseases such as Alzheimer’s disease [2], while large cohort studies demonstrated that short (5 h or less) and long sleepers (more than 9 h) live shorter than people with an appropriate amount of sleep (6 to 8 h per night) [3,4,5]. In electroencephalogram (EEG) recordings, there are two distinct stages characterizing sleep: non-rapid eye movement (NREM) sleep, sometimes also called slow wave sleep, and rapid eye movement (REM) sleep or paradoxical sleep. NREM sleep is characterized by slow but relatively high-amplitude oscillations, while REM sleep exhibits an EEG with higher frequency but lower amplitude, similar to (but distinct from) that of wakefulness. Sleep has an essential function to allow the human body to physically restore and heal itself. It is especially important to maintain an efficient immune system and avoid metabolic and cardiovascular disorders associated with insomnia. In the brain, sleep is also essential in some memory consolidation processes [6] and possibly for brain detoxification [7,8].

The regulation of sleep and wakefulness involves many regions and cellular subtypes in the brain. Indeed, the ascending arousal system promotes wakefulness through a network composed of the monaminergic neurons in the locus coeruleus (LC), histaminergic neurons in the tuberomammilary nucleus (TMN), glutamatergic neurons in the parabrachial nucleus (PB) and orexinergic neurons in the lateral hypothalamus, among others. On the other hand, only a handful of regions able to promote sleep have been identified so far. The ventrolateral pre-optic area (VLPO) was the first “sleep center” to be identified by Saper’s team [9], and is considered as the master regulator for the so-called “wake/sleep flip-flop switch” [10]. More recently, Fuller’s laboratory also discovered that sleep can be promoted by the activation of a gamma-aminobutyric acid-ergic (GABAergic) population of neurons located in the parafacial zone [11,12], while the role of the GABAergic A_2A_R-expressing neurons of the nucleus accumbens [13] and the striatum has just been revealed [14,15]. In total, more than 50 neurotransmitters and their respective receptors are involved in the process of controlling the vigilance state of the brain.

## 3. Zinc and the Central Nervous System

The trace metal zinc is an essential cofactor for more than 300 enzymes and 1000 transcription factors [16]. A moderate deficiency of zinc is sometimes observed in humans, and is responsible for growth retardation, male hypogonadism, taste alteration, inefficient wound healing and immune system, as well as mental retardation. In the central nervous system, zinc is the second most abundant trace metal and is involved in many processes. In addition to its role in enzymatic activity, it also plays a major role in cell signaling and modulation of neuronal activity. Zinc finger proteins, a huge family of zinc-containing proteins, play key roles in the mechanisms of DNA replication and transcription regulation [17,18,19,20]. Zinc has also been implicated in neurodegenerative diseases. Some Alzheimer’s disease patients exhibit a systemic deficiency in zinc [21], however, it has also been proven that amyloid plaques are highly enriched in zinc. It is possible that the amyloid plaques immobilize the pool of zinc in the brain and therefore reduce the bioavailability into the neurons.

Zinc is utilized by tissue as a function of zinc transporters. Zinc transporters are playing an important role in the homeostasis of zinc and are tightly controlling concentration of this ion in the different organs in order to allow proper biological functions, while impaired zinc transporter function correlates with clinical human diseases [22]. Interestingly, a research in Drosophila studied the molecular polymorphisms of the gene *Catecholamines up*, strongly associated with day sleep [23], and characterized it as the Drosophila ortholog of the mammalian *ZIP7* zinc transporter [24].

In addition to its role as a cofactor, zinc is also a modulator of neuronal activity in the brain. While the majority of zinc is protein-bound, some specific subpopulations of neurons contain vesicles filled with weakly bound or free zinc ions (Zn^2+^). These zinc-containing neurons were first identified in the mossy fibers of the hippocampus [25]. The first reported and most abundant population of zinc-containing neurons is glutamatergic (sometimes called “gluzinergic” neurons), and zinc released from the vesicles of these neurons into the synaptic cleft could modulate *N*-methyl-d-aspartate receptor (NMDAR) activity in a dose-dependent and reversible manner [26,27,28]. However, zinc can also modulate the activity of other glutamate receptors, such as α-amino-3-hydroxy-5-methyl-4-isoxazolepropionic acid (AMPA) [29], metabotropic receptors [30] as well as the receptors for other neurotransmitters [31] such as adenosine [32], dopamine [33] and serotonin [34]. Furthermore, zinc can also decrease the uptake of glutamate [35] and dopamine [36] transporters, and it exhibits various effects on calcium [37], potassium [38], sodium [39] and chloride [40] channels, while recent evidence demonstrates that zinc can also be released into glycinergic synapses [41].

One of the best-characterized physiological functions of zinc after its release into the synaptic cleft involves the modification of hippocampus-dependent memory by the amygdala. The lateral nucleus of the amygdala, a component of the limbic system that is essential for emotion, receives massive projections from the entorhinal cortex. Kodirov et al. suggested that synaptically released Zn^2+^ in that location was responsible for long-term potentiation (LTP) by depressing feed-forward GABAergic inhibition of the post-synaptic neurons and thus serves as an essential mechanism for the acquisition and storage of spatial memory in a learning task [42].

For a long time, measuring zinc concentration in the synaptic cleft remained approximate at best, and reported results could vary by several orders of magnitude [43]. Finally, a recent in vivo study determined that the resting concentration of synaptic zinc is extremely low (<10 nM), but after being released from pre-synaptic vesicles after ischemic stroke zinc concentration rises quickly (within a few milliseconds) and remains in the nanomolar range, which is sufficient to activate high-affinity receptors (such as NMDA Receptor 2A “GluN2A”) but not low-affinity receptors [44].

After zinc has been released from gluzinergic neurons into the synaptic cleft, its concentration is rapidly decreased through several mechanisms. First, a very efficient mechanism of zinc reuptake is in charge to remove available zinc and reconstitute zinc vesicles [44]. Second, unlike conventional neurotransmitters, zinc can be translocated from the synaptic cleft (or even the pre-synaptic vesicle) into post-synaptic neurons through zinc-permeable gated channels such as NMDAR [45]. Furthermore, an unknown amount of zinc is expected to diffuse away from the synaptic cleft due to the concentration difference between the cleft and the cerebrospinal fluid (CSF). It is also hypothesized that glial cells play a critical role not only in the removal of released zinc but also in the integration of synaptic transmission modulated by zinc [46]; however, the precise mechanisms remain elusive.

## 4. “Sleep as You Eat” or How Food Can Regulate Sleep

Eating and sleeping are two intrinsic essential activities in animals. Numerous studies have reported how our sleep status can modulate the way we eat. For example, total sleep deprivation for one night in healthy adults resulted in an increase of desire for highly palatable food compared to control non-sleep deprived subjects [47]. Even partial but chronic sleep deprivation was sufficient to increase food consumption beyond the physiological balance and induce weight gain [48]. Interestingly, recent experiments on mice demonstrated that a partial inhibition of REM sleep increased the absorption of high-calorie food; however, blocking neuronal activity in the medial prefontal cortex could reverse the effect on sucrose but not fat consumption [49]. On the other hand, a growing number of studies has reported the opposite effect, where diet regulates sleep [50,51,52]. In a pioneering clinical study, Phillips et al. provided an isocaloric high carbohydrate/low fat (HC/LF) or low carbohydrate/high fat (LC/HF) diet to eight healthy young men. They observed a significant decrease of NREM sleep after consumption of the HC/LF diet, and an increase of REM sleep for both diets compared to a balanced control diet [53].

Not only the quantity of sleep, but also its quality can be modulated by our eating behavior. A recent study demonstrated the surprising beneficial effects of kiwifruit consumption on sleep quality in a four-week trial on 24 subjects. The score of the Chinese version of the Pittsburgh Sleep Quality Index (CPSQI) auto-evaluation test, the waking time after sleep onset, as well as the sleep onset latency were significantly decreased, while the total sleep time and the sleep efficiency were significantly increased [54]. While the mechanisms involved in such effects remain elusive, recent efforts permit the identification of active compounds and their molecular mechanisms in sleep-promoting foods and natural compounds including saffron [55,56], honokiol [57], magnolol [58], phlorotannin [59], sake yeast [60,61] or ashwagandha leaf extracts [62]. Finally, Grandner et al. analyzed data from the National Health and Nutrition Examination Survey on 5587 American citizens to determine the dietary nutrients associated with short and long sleep duration [63]. They identified several vitamins and minerals whose dietary intake correlated with a modification of sleep amount, and notably characterized zinc as one of them. According to their results, very short sleepers (<5 h) ingested significantly less zinc than did normal or long sleepers. These results are in accordance with the very limited number of studies that have compared zinc amount in humans and sleep patterns. The difference of zinc consumption observed in this study might be the result of the consumption of food more or less rich in zinc between subjects such as oyster, other seafood and meat. It would also be interesting to measure to which extent blood-zinc concentration correlates with zinc consumption and to determine if this parameter also varies with the amount of sleep.

## 5. Sleep Regulation, an Unexpected Function of Zinc

### 5.1. Clinical Studies

In 2009, a population study on 890 healthy Jinan residents in China evaluated the relationship between zinc/copper serum concentrations and several physiological factors such as sex, age, drinking and smoking behavior, and sleep [64]. Regarding sleep, the mean concentration of serum copper remained constant regardless of the amount of sleep; however, the highest concentration of serum zinc was found in subjects sleeping a “normal” amount of 7 to 9 h per night (1.337–1.442 mg/L), compared to short (<7 h) and long (>9 h) sleepers (0.789–0.934 mg/L). A later cross-sectional study measured zinc and copper content in the serum and hair of 126 adult Korean women [65]. This time, the group of women with the highest serum and hair zinc/copper ratio had the highest percentage of optimal amount of sleep (7–7.9 h). Recently, another Chinese cohort study compared blood zinc concentration and sleep quality in 1295 children from the Jintan Child Cohort [66]. Blood sampling was performed on the same children twice: at preschool (3–5 years old) and several years later during their 6th grade (11–15 years old). No significant association between zinc status and sleep could be found in these children in their younger age; however, blood zinc concentration correlated with sleep duration and sleep quality (CPSQI test) in their pre-adolescent age. Furthermore, a longitudinal association between the first and second sampling periods demonstrated that zinc blood concentration at preschool age predicted the development of poor sleep quality and efficiency several years later.

**Figure 1:**
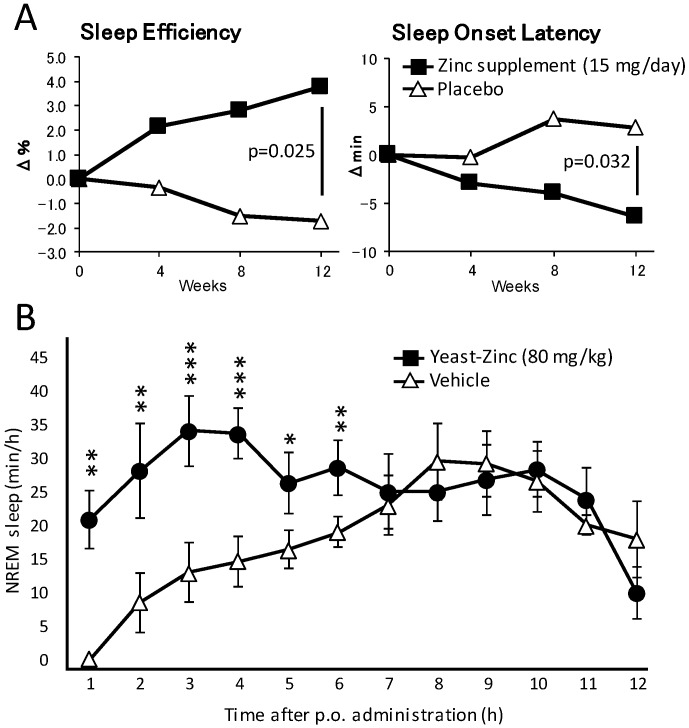
Dietary zinc improves sleep quality in humans and increases NREM sleep in mice. (**A**) Two groups of 30 volunteers absorbed daily 15 mg of zinc (in 40 g of Pacific oysters) or placebo (40 g of scallops). After 12 weeks of supplementation, sleep efficiency and sleep onset latency improved in the group treated with zinc compared to the control group. (**B**) Oral administration of zinc-containing yeast extract (80 mg/kg) in mice at the onset of dark time increased the amount of NREM sleep for 6 h compared to mice receiving vehicle. * *p* < 0.05, ** *p* < 0.01, *** *p* < 0.001 compared with vehicle treatment.

As well as these reports, to the best of our knowledge only three studies have more or less directly evaluated the effect of zinc supplementation on sleep in humans. In the first study, the authors focused on the effect of a 12-month iron and zinc supplementation on sleep in 877 infants from Zanzibar and 567 infants from Nepal, both groups being vastly subjected to malnutrition [67]. Infants from Zanzibar not suffering from iron deficiency anemia (IDA) and who received supplemental zinc slept an extra 1.3 h at night and a total of 1.7 h extra per day (night sleep + naps) compared to infants receiving a placebo. Zinc supplementation also resulted in sleep time increase in Nepalese infants with IDA, albeit to a smaller extent. In another double-blind placebo-controlled clinical trial, the authors evaluated the effect of a triple supplementation of melatonin, magnesium and zinc on 43 residents of a long-term care facility in Italy who exhibited primary insomnia [68]. Patients ingested daily a combination of melatonin (5 mg), magnesium (225 mg) and zinc (11.25 mg) mixed in 100 g of pear pulp for 60 days, one hour before bedtime. Patients that received this mineral supplement exhibited a remarkable improvement of sleep quality with a Pittsburgh Sleep Quality Index reduced from 12.7 ± 2.6 to 5.5 ± 1.9. On the other hand, placebo-treated patients did not show any sleep quality improvement (12.3 ± 3.6 and 12.0 ± 4.4, respectively, before and after placebo treatment). Finally, the most recent study determined the effect of zinc supplementation from natural sources (zinc-rich oysters and zinc-containing yeast extracts) on 120 healthy subjects in a randomized controlled trial in Japan [69]. Compared with the placebo group, individuals treated for three months with daily zinc supplements demonstrated an improved sleep onset latency and sleep efficiency compared to control subjects (Figure 1A).

### 5.2. Experimental Evidence

All these human studies measure the amount or the quality of sleep in correlation with zinc supplementation, and conclude with an improvement of the sleep pattern of the tested subjects. However, because zinc is not provided alone, the effects might arguably arise from different compounds administered at the same time as zinc. For instance, zinc supplementation was complemented with iron in the Zanzibar and Nepal study, while clinical examination demonstrated iron deficiency in a substantial amount of the children before the beginning of the study. Providing iron to IDA infants might account for the improvement of sleep observed by the authors. In the Italian study, zinc was complemented with melatonin and magnesium; however, melatonin’s effect on sleep patterns has been extensively studied and demonstrated [70] and it is unfortunate that the authors did not decipher how important each component was on sleep. Similarly, oysters contain a large amount of taurine, a γ-aminobutyric acid (GABA) receptor agonist known to promote sleep-like resting behavior in *D. melanogaster* [71]. Furthermore, environmental factors as well as emotional condition also have a major impact on how well and how long humans sleep, and can interfere with zinc’s effects on sleep.

The most convincing study to date about the effect of zinc on sleep was obtained in our study published in 2015 [72]. In these experiments, we used a mouse model, which eliminates all the environmental and psychological factors that might have negatively influenced the previous human studies. Feeding the mice with zinc-containing yeast extract (equivalent to a dose of 10 to 160 mg/kg of elemental zinc) at the onset of the dark phase resulted in a drastic reduction of locomotor activity for a period of up to 6 h. Such an effect could not be observed if the mice were fed with a similar amount of yeast extract rich in the other divalent cations manganese, iron or copper, proving a specific effect for zinc. We could also precisely measure and characterize the zinc-induced sleep by recording the EEG and its power spectrum during the experiment. Yeast-zinc, orally administered at a dose of 40 or 80 mg/kg, dose-dependently and specifically increased the total amount of NREM sleep when administered at the onset of the dark phase, when the animal is most active (Figure 1B). Furthermore, the power spectrum of NREM sleep remained indistinguishable from that of physiological NREM sleep, demonstrating a good sleep quality.

However, the same doses of zinc-containing yeast extract had no significant effect on the amount of sleep when administered during daytime, when the animal is already mostly sleeping. Under basal conditions, a mouse sleeps an average of 20 min/h during nighttime and 40 min/h during daytime. When mice were fed with zinc at the onset of the dark phase, the total amount of sleep increased by up to 20–30 min/h, but, when they were fed during daytime, it remained at around the usual 40 min/h. In other words, zinc never induced sleep beyond the physiological level, contrary to more classical sleep-inducing molecules such as benzodiazepine, which also reduce the power density of NREM sleep and result in poor sleep quality. It is possible that zinc acts on circadian regulators and induces sleep when the animal is usually sleeping.

## 6. Solving the Mystery of Zinc-Induced Sleep

One may wonder how dietary zinc might act so quickly on the central nervous system (CNS) and regulate a function as essential as sleep. It is well accepted that the blood–brain barrier (BBB) has a very low permeability for zinc and the concentration of this ion remains extremely stable in the CSF regardless of serum zinc concentration [73]. However, a higher time-resolution measurement in rats revealed a rapid exchange of zinc between blood and brain during the first 30 min following intravenous administration, and zinc was not stored in the CSF but in an undetermined compartment of the brain [74,75]. A later study demonstrated the variable permeability of the BBB for zinc using an in vitro experimental model [76]. In this experiment, the BBB exhibited a constant zinc permeability when the plasma compartment concentration remained between 10 and 25 µM; however if this concentration increased further, BBB permeability for zinc increases suddenly and markedly. In our experiments, we found that zinc concentration increased in the serum up to 10-fold after the oral administration as compared to baseline. We therefore hypothesize that orally administered zinc reaches some specific compartment of the CNS after rapidly increasing in the blood, thus activating a signaling pathway that is responsible for the promotion of sleep. It would be unrealistic to think that, in physiological conditions, dietary zinc could be responsible for regulating sleep in animals and humans, especially since it seems unlikely that it would affect the concentration of zinc in the CNS. However, local zinc concentration may be less stable in the CNS than one would expect. Indeed, it was also reported that plasma zinc concentration, while being tightly controlled by diverse mechanisms, exhibits a circadian variation, with a minimum concentration measured in the evening while it reaches its peak in the morning [77]. Similarly, we hypothesize that the zinc concentration also varies in some specific regions of the CNS during the course of a day.

The idea that ions can regulate wake and sleep is actually not new. In 1927, Demole discovered that the injection of CaCl_2_ into the pituitary of cats increased sleep for several hours. More recently, Nedergaard’s lab thoroughly studied the interaction between sleep/wake status and the CSF concentration of three ions: K^+^, Ca^2+^ and Mg^2+^ [78]. They reported that the concentration of extracellular K^+^ increases in the CSF of mice during wakefulness, while those of Ca^2+^ as well as Mg^2+^ decreases. Moreover, the authors assessed whether the sleep/wake status was responsible for the change in ion concentrations or if the change in ion concentrations was physiologically responsible for the promotion of sleep and wakefulness. They demonstrated that infusion of artificial CSF mimicking the concentration of ions in wake or sleep reversed the neuronal activity and the behavioral state. Involvement of Ca^2+^ in the control of sleep was also recently demonstrated in mammals [79]. These results shed new light on the importance of the non-neurotransmitter type of communication within the brain, involving ions such as Ca^2+^, Mg^2+^ and also Zn^2+^, to control even the most critical physiological functions.

The specific mechanism of action of zinc in the CNS to promote sleep remains elusive. Most studies looking at the activity of Zn^2+^ in the brain have focused on its interaction with the glutamatergic receptors, because Zn^2+^ exists predominantly in the presynaptic vesicles of glutamatergic neurons to be co-released with glutamate. Some other receptors also interact with zinc. Zn^2+^ is also found not only in glutamatergic axon terminals but also in some inhibitory axon terminals, potentially glycinergic, of the cerebellum and in the spinal cord [80]. Glycinergic neurons project to orexin neurons in the lateral hypothalamus (well characterized neurons that are involved in the maintenance of wakefulness), and can inhibit their activity [81]. The glycinergic receptor (GlyR) exhibits an atypical reaction in the presence of zinc. First, the synaptic activity of the α1β GlyR isoform is increased in vitro even in the presence of a very low concentration of zinc (10 nM–1 µM). However, at a higher concentration (3–300 µM), zinc exhibits a dose-dependent inhibition of GlyR excitability. A recent in vivo study revealed that the free Zn^2+^ concentration in the glycinergic synaptic cleft could rises to at least 1 µM following a single presynaptic stimulation, which is higher than the concentration in glutamatergic synapses [41]. Furthermore, selective mutation in the α1 subunit of the glycine receptor identified Zn^2+^ as an essential endogenous modulator of glycinergic transmission, leading to the development of a hyperekplexia phenotype in mice, and demonstrated that zinc is essential for the proper functioning of the glycinergic system [82]. However, it remains unclear whether zinc is released directly from presynaptic glycinergic neurons or from neighboring zinc-containing glutamatergic neurons.

As well as acting as a cofactor for various receptors, zinc has its own receptor called G protein-coupled receptor 39 (GPR39) [83], which is a G_αs_ protein-coupled receptor to activate adenylate cyclase and cAMP-dependent signaling. In the brain, it is mainly expressed in the amygdala, the hippocampus and the auditory cortex, as well as in many other regions to a lesser extent [84]. The administration of zinc to hippocampal slices activated GPR39 in the CA3 region and regulated neuronal activity by inducing intracellular release of calcium, as well as phosphorylation of extracellular-regulated kinase and Ca^2+^/calmodulin kinase II [85]. Deletion of GPR39 in mice led to the development of a depression-like behavior [86,87] as well as an increased risk of Alzheimer’s disease [88], two well-characterized pathologies related to sleep disturbance [89]. To the best of our knowledge, nobody has yet checked the potential involvement of GPR39 in the regulation of sleep and wakefulness, let alone the potential role of zinc in this physiological process.

## 7. Conclusions

The role of zinc in the CNS has become increasingly important, since we first recognized its central role in the regulation of essential functions such as memory and, now, sleep. However, much work remains to comprehend properly the key functions of zinc in glutamatergic transmission and other types of neurotransmission. Although zinc ion was of little interest to the scientific community for a long time, accumulating evidence proves that endogenous zinc as well as available dietary zinc is of high importance, not only as an enzyme cofactor but also as a signaling molecule. One of the most unexpected functions of zinc to date may be in the regulation of sleep, an essential physiological function shared by the entire animal kingdom. While the mechanisms by which zinc regulates sleep remain unclear, rapid progress towards their elucidation is to be anticipated.

## Abbreviations

CNS	central nervous system
EEG	electroencephalogram
NREM	non-rapid eye movement
REM	rapid eye movement
LC	locus coeruleus
TMN	tuberomammillary nucleus
PB	parabrachial nucleus
VLPO	ventrolateral pre-optic area
DNA	deoxyribonucleic acid
GABA	γ-aminobutyric acid
NMDAR	*N*-methyl-d-aspartate receptor
AMPA	α-amino-3-hydroxy-5-methyl-4-isoxazolepropionic acid receptor
HC	high carbohydrate
LF	low fat
LC	low carbohydrate
HF	high fat
CPSQI	Chinese version of the Pittsburgh Sleep Quality Index
IDA	iron deficiency anaemia
BBB	blood–brain barrier
GlyR	glycinergic receptor
GPR39	G protein-coupled receptor 39
p.o.	per os
